# Intervertebral Disc Proteoglycans: Multifunctional Tissue Stabilizing and Instructional Cell Regulatory Proteins That Control Tissue Homeostasis

**DOI:** 10.1002/jsp2.70145

**Published:** 2025-12-09

**Authors:** James Melrose

**Affiliations:** ^1^ Raymond Purves Bone and Joint Research Laboratory Kolling Institute St. Leonards New South Wales Australia; ^2^ School of Medical Sciences, Faculty of Medicine and Health The University of Sydney at Royal North Shore Hospital St. Leonards New South Wales Australia; ^3^ Graduate School of Biomedical Engineering, Faculty of Engineering University of New South Wales Sydney New South Wales Australia

**Keywords:** aggrecan, bikunin, intervertebral disc, lubricin, matricryptins, perlecan

## Abstract

The intervertebral disc (IVD) is a tough fibrocartilaginous viscoelastic cushion interposed between spinal vertebrae. It has a composite structure whose material properties are due to the interplay between fibrillar collagens which, provide high tensile strength and hydrodynamic IVD proteoglycans (PGs) such as aggrecan that provide weight‐bearing properties. Other PGs such as the small leucine‐rich PGs, have roles in collagenous assembly and organization and interactions with growth factors and cytokines. Elastin in the annular lamellae provides resilient properties to tissues. A total of 19 IVD PGs have been identified; three recently identified PGs, perlecan, bikunin and lubricin have particularly interesting properties that affect tissue function. Perlecan is a multifunctional HSPG that provides tissue stabilization and promotes cellular proliferation and differentiation during spinal development, IVD repair and cell‐extracellular matrix communication as well as osmoregulatory properties allowing the disc cell to sense and respond to its biomechanical microenvironment to maintain tissue function and regulate IVD homeostasis. Bikunin, the light chain of inter‐α‐trypsin inhibitor (ITI) has tissue protective properties and roles in the transfer of the heavy chains of ITI to hyaluronan which, aids in its stabilization. Lubricin is a boundary lubricant identified in the surface regions of articular cartilage but its roles in the IVD have yet to be fully determined. It may aid in the lubrication of collagen fiber bundles in the annulus fibrosus allowing them to slide over each other during torsional loading. Emerging roles for lubricin in cell regulation and control of inflammation may also be relevant to IVD regulatory processes. This review illustrates the diverse structure and function of IVD PGs and their roles in cellular regulation and tissue function. Fragmentation of IVD PGs during normal tissue turnover generates some interesting bioactive PG fragments of potential application in tissue repair and regenerative processes that warrant further investigation.

## Graphical abstract

Matrix and cell‐associated proteoglycans are important tissue‐stabilizing, weight‐bearing, and tension‐resisting proteins in the intervertebral disc. Their glycosaminoglycan components have growth factor binding and cell‐instructive properties that allow cells to regulate tissue composition and function.

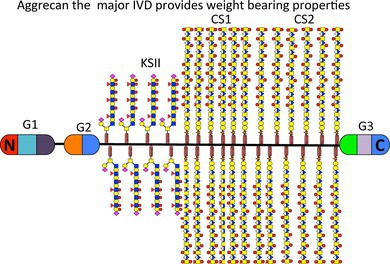

**Abbreviations**
ACAN: aggrecan
ADAMTS4: a disintegrin and metalloproteinase with thrombospondin motifs 4
AF: annulus fibrosus
Akt1: RAC‐alpha serine/threonine‐protein kinase B
ASPN: asporin
BGN: biglycan
BKS‐1(+): a keratan sulfate neoepitope generated by keratanase pre‐digestion
CAPAGE: composite agarose‐polyacrylamide gel electrophoresis
CEP: cartilaginous endplate
CHAD: chondroadherin
DCN: decorin
ECM: extracellular matrix
EGF: epidermal growth factor
ERK: extracellular signal‐regulated kinase
FAK: focal adhesion kinase, a nonreceptor cytoplasmic tyrosine kinase
FMOD: fibromodulin
GAG: glycosaminoglycan
GF: fibroblast growth factor
GPC: glypican
HA: hyaluronan
HLE: human leucocyte elastase
HNK‐1: human natural killer trisaccharide, HSO_3_‐3GlcAß1‐3Galß1‐4GlcNAc‐
HSPG2: perlecan
HYAL4: hyaluronidase‐4, a CS hydrolase
IGF‐1: insulin‐like growth factor‐1
IL: interleukin
ITI: inter‐alpha‐trypsin inhibitor
ITI‐HC: inter‐alpha trypsin inhibitor heavy chain
IVDD: intervertebral disc degeneration
JAK2: Janus kinase 2
KERA: keratocan
KPI: Kunitz protease inhibitor domain
KS: keratan sulfate
LBP: low back pain
LRR: leucine rich repeat
LUM: lumican
MAb: monoclonal antibody
MAPK: mitogen‐activated protein kinase
MMPs: matrix metalloproteases
MSCs: mesenchymal stem cells
NFκB: nuclear factor kappa B
NP: nucleus pulposus
OGN: osteoglycin
OMD: osteomodulin
OMOD‐1: omodysplasia
PCM: pericellular matrix
PRELP: prolargin
PRG4: proteoglycan 4, lubricin
PSGL‐1: P‐selectin glycoprotein ligand‐1
rhADAMTS‐4,5: recombinant human ADAMTS‐4, 5
ROS: reactive oxygen species
SDC4: syndecan‐4
SDS: sodium dodecyl sulfate
SHH: Sonic hedgehog
SLRP: small leucine rich proteoglycan
SMA: smooth muscle cell actin
SPI: serine protease inhibitor
TGFβ: transforming growth factor beta
TLR: toll‐like receptor
TNFα: tumor necrosis factor alpha
TSP‐1: thrombospondin‐1
VCAN: versican
VGP: vertebral growth plate
VLDL: very low density lipoprotein
Wnt: wingless‐related integration site

## 1 Introduction

Extracellular matrix (ECM) proteoglycans (PGs) are important functional components of the intervertebral disc (IVD), the largest avascular and aneural structure in the human body. This equips the spine with the ability to withstand axial compressive loading and also provides flexibility in lateral, forward and backward bending, torsion, flexion and extension. A total of 13 IVD PGs have been identified from proteomic studies of human and animal IVD models in health and disease [1, 2, 3, 4], and an additional 6 PGs have been identified in further studies [5, 6, 7, 8, 9, 10] making a total of 19 IVD PGs (Table 1). This review illustrates the structural diversity of IVD PGs and their functional properties in the complex IVD composite structure.

**TABLE 1:** Intervertebral disc proteoglycans identified in proteomic studies (A) and in biochemical characterization studies (B).

(A) IVD proteoglycans identified in proteomic studies [1, 2, 3, 4]	
Gene	Proteoglycan
ACAN	Aggrecan
ASPN	Asporin
BGN	Biglycan
CHAD	Chondroadherin
DCN	Decorin
FMOD	Fibromodulin
HSPG2	Perlecan
LUM	Lumican
OGN	Osteoglycin
OMD	Osteomodulin
PRELP	Prolargin (proline and arginine rich end leucine rich repeat protein)
PRG4	Lubricin
VCAN	Versican

(B) The full spectrum of IVD proteoglycans and their functional roles in health and disease		
Proteoglycan	Proteoglycan features	References
Asporin	ASPN, Asporin levels are elevated in IVDD and is an important risk factor, the Asporin D14 allele is associated with IVDD. Asporin is a 39 kDa SLRP (small leucine rich PG) containing 14 unique aspartic acid (D) residues in its N‐terminus, otherwise it is closely related to DCN and BGN. ASPN inhibits chondrogenesis in vitro by down‐regulation of TGF‐β signaling leading to the susceptibility to development of IVDD.	[11, 12, 13, 14]
Aggrecan	ACAN, aggrecan forms aggregates with HA that are important in the hydration of IVD tissues and in the generation of an internal hydrostatic pressure in the NP which, equips the IVD with viscoelastic hydrodynamic properties which, allows the IVD to withstand axial spinal loading. Aggrecan is targeted by a number of MMPs and ADAMTS 4 and 5 during IVDD leading to its depletion in the NP and a significant reduction in IVD biomechanical competence. Degenerated IVD tissues depleted of aggrecan become susceptible to an ingrowth of blood vessels and nerves into the IVD. Cells within the biomechanically incompetent degenerate IVD become mechano‐sensitized leading to the generation of LBP.	[15, 16, 17, 18, 19, 20]
Versican	VCAN, versican occurs in several alternatively spliced iso‐forms including full‐length versican (Vo isoform), V1 (no GAG−α domain) and V2 (no GAG−β domain) isoforms. The V3 versican isoform contains G1 and G3 domains but no GAG−α and GAG−β domains. The G1 and G3 domains promote cellular migration involving epidermal growth factor‐like (EGF) domains. Versican is a component of the AF and NP and associates with elastic networks that attach adjacent lamellar layers and may contribute to the cohesivity of annular lamellae in the composite disc structure. The CS chains of versican in fetal human IVDs are labeled with monoclonal antibody (MAb) 7D4 which, is absent in adult tissues and is a biomarker of tissue morphogenesis.	[21, 22, 23, 24, 25]
Perlecan	HSPG2, perlecan has prominent chondrogenic properties in the development of the cartilaginous fetal spinal column and roles in the formation of ossification centers in the vertebral rudiments which, lead to bone formation. Perlecan promotes development of the AF, NP and CEPs in tissue morphogenesis and has multifunctional roles in tissue stabilization through interactions with HS binding structural glycoproteins, its localization pericellularly and interaction with type VI collagen protects IVD cells from overloading. Perlecan provides mechanoresponsive and osmoregulatory properties to IVD cells, interactive instructional cell‐ECM links and aiding in tissue homeostasis. Perlecan aids in ECM stabilization and tissue repair.	[26, 27, 28, 29, 30, 31, 32, 33, 34, 35]
Collagen XVIII	Collagen XVIII exists as long intermediate and short alternatively spliced forms and forms networks with perlecan in blood vessel basement membranes. Collagen XVIII's stabilizing roles in the IVD only become apparent when mutations lead to a deficiency of this PG in the IVD.	[36, 37, 38]
The Syndecan (SDC) family	SDC4, syndecan‐4 is expressed by NP cells and is implicated in the maintenance of physiological functions in the IVD and the establishment of the lamellar structure of the IVD. SDC4 performs various functions, including cell–cell and cell‐ECM interactions, growth factor–receptor activation, matrix adhesion and MMP activation. SDC2 and SDC4 may act collectively to maintain IVD homeostasis, however SDC4 is implicated in IVDD mediating matrix degradation by controlling the activity of ADAMTS5 and MMP3.	[39, 40, 41, 42, 43, 44, 45, 46, 47, 48, 49, 50]
The glypican (GPC) family	GPC6 polymorphisms are associated with lumbar disk herniation risk. Dysfunctional PGs such as GPC6 represent a functional link between IVDD and skeletal dysplasia.	[51, 52, 53]
Decorin	DCN, decorin regulates a wide range of biological processes effecting cell growth, differentiation, proliferation, adhesion, spread and migration, and regulates inflammation, inhibits TGF‐β, controlling tissue fibrosis and ECMcollagen remodeling. DCN levels are elevated in early stages of IVDD but it becomes fragmented in later stages of IVDD.	[54, 55, 56, 57, 58, 59, 60, 61]
Biglycan	BGN, biglycan regulates subchondral bone structure and regulates type I and II collagen fibrillogenesis, interacts with type VI collagen and the assembly of elastin fibers contributing to ECM stabilization. BGN also binds to dystroglycan modulating cell signal transduction during cell growth and differentiation. TGF‐β1 is the most pro‐fibrotic cytokine, and its activity in vivo is related to BGN levels. Targeting of BGN by MMPs may represent a useful strategy to selectively modify tissue material properties.	[62, 63, 64, 65, 66, 67]
Lumican and Fibromodulin	The LRRs of FMOD and LUM interact with an extensive range of ECM proteins that organize and stabilize tissue form and function. FMOD and LUM reciprocally regulate collagen fibrillogenesis and do not share functional equivalence. FMOD promotes formation of thick collagen fibers whereas LUM forms thin collagen fibers. N‐terminal sulfated tyrosine residues interact with FGF‐2, TSP‐1, MMP‐13, NC4 domain of collagen IX, and IL‐10, bind to collagen and promote fibril formation. FMOD sequesters TGF‐β controlling its bio‐availability, binds C1q and activates the complement system. LUM impedes tumor growth through its MMP‐14 inhibitory activity, affects focal adhesions and the migration and growth of tumor cells through interaction with α2β1 integrin and inhibitory effects on angiogenesis. LUM regulates bacterial LPS TLR‐4 endotoxin interactions with CD14, promoting bacterial phagocytosis in innate immunity and inflammation. FMOD and LUM are degraded in an ovine model of IVDD. LUM has been immunolocalised in rat IVDs using KS neo‐epitope MAb BKS‐1 (+).	[60, 61, 68, 69, 70, 71, 72, 73, 74, 75, 76, 77, 78]
Keratocan	KERA, keratocan originally isolated from cornea is a 50 kDa IVD SLRP that has been immunolocalised in IVD and spinal cord. Keratocan contains low sulfation KS‐I side chains and has neuroregulatory and matrix organizational roles during spinal development mediated by growth factor and morphogen interactions. Keratocan is cleaved into two prominent fragments during IVDD.	[10, 60, 79]
Prolargin, PRELP	PRELP shares 36% identity with FMOD, and 33% with LUM, anchors perlecan and type I collagen to basement membranes, and type II collagen to cartilage. PRELP has an N‐terminal Arg and Pro extension, its alternative name is prolargin.	[80, 81]
Chondroadherin	CHAD, chondroadherin is a 38 kDa member of the KS‐SLRP family containing 11 LRRs that bind to α2β1 integrin, type I, II and VI collagen and has an anchoring role in ECM stabilization, binds cells to the ECM and mediates cell‐ECM communication through interactions with cell surface PGs such as the syndecans.	[82, 83, 84, 85, 86]
Osteomodulin	OMD, Osteomodulin (osteoadherin) is a KS‐SLRP that binds to osteoblasts via αVβ3 integrin and regulates osteogenesis through its interaction with BMP2. WNT1 transcriptionally activates the expression of OMD.	[87, 88, 89]
Osteoglycin	OGN, Osteoglycin (mimecan) is a class II SLRP with diverse roles in ECM assembly, regulates bone formation along with TGF‐β1/TGF‐β2 to control collagen fibrillogenesis and also has regulatory roles in metabolic health, cancer and diabetes regulating glucose homeostasis.	[90, 91, 92, 93, 94, 95, 96]
Serglycin	SRGN, Serglycin is a small molecular weight intracellular heparin‐PG that can also be secreted and incorporated into the ECM by IVD cells. SRGN levels are elevated in inflammatory conditions during IVDD. IL‐1β or TNF‐α increase SRGN expression in vitro. SRGN stores key inflammatory mediators in an inactive form in storage granules and secretory vesicles forming intracellular complexes with serine proteases (elastase, chymase, tryptase, carboxypeptidase).	[8, 97, 98, 99, 100, 101, 102, 103, 104, 105, 106]
Lubricin, PRG4	Lubricin, originally identified as a boundary lubricant in articular cartilage has emerging roles as an active anti‐inflammatory molecule with diversified functions in a number of tissues. Lubricin's hemopexin domain has a scavenging role for free heme which, is highly toxic, is a novel ligand for CD44 and may control HA interactions with Toll‐like receptors (TLR's) during inflammation. Lubricins precise role in the IVD is not known but its localization in specific tissue compartments in flexor tendon, rotator cuff and IVD suggests it may facilitate the sliding of collagen fiber bundles in fascicles and in annular lamellae during torsional loading.	[107, 108, 109, 110, 111, 112]
ITI/Bikunin	Bikunin, the light chain of ITI is a 25–26 kDa CSPG with two Kunitz protease inhibitor (KPI) domains. The CS chain of bikunin contains CS‐A (2B6) and embedded CS‐D (MO‐225) disaccharides and non sulphated IB5 chondroitin. The KPI domains have potent inhibitory activity against, leucocyte elastase, cathepsin G, trypsin, chymotrypsin, kallikrein. Bikunin also has anti‐viral properties.	[5, 22, 113]

Despite their importance to tissue function, full‐time PGs only represent a small percentage of the total number of proteins encoded in the human genome [114]. Of 20 000 protein‐encoding genes in the main human databases, (GENCODE/Ensembl, RefSeq, and UniProtKB) only ~50 PGs have been identified representing ∼0.25% of all proteins. The core proteins of PGs are highly conserved across species pointing to their functional importance [115]. Full‐time PGs are those in which, all glycosaminoglycan (GAG) substitution sites are permanently occupied on the core protein in contrast to part‐time PGs where this may not be the case. Part‐time PGs can occur as GAG free isoforms in some tissue contexts. Examples include neuroglycan C [116], CD44, macrophage colony stimulating factor and decorin [117]. The term part‐time proteoglycan, was introduced by Ruoslahti in 1989 [118].

## 2 Glycan Sequence and Charge Heterogeneity Provides Diverse Interactivity of GAG Side Chains of PGs With a Large Range of Ligands

**FIGURE 1:**
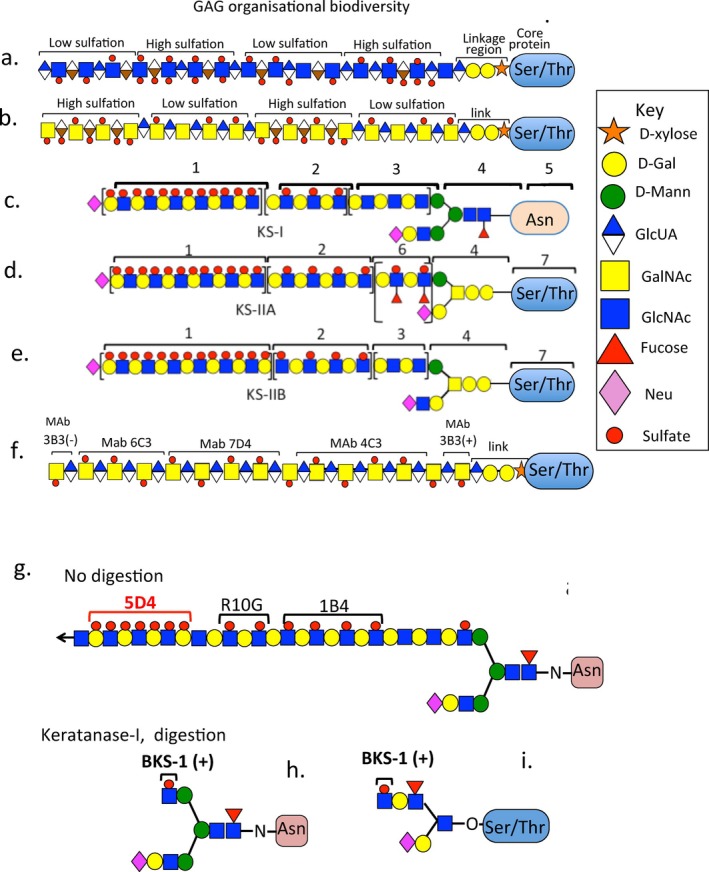
The complexity of proteoglycan GAG side chains showing the variable sequences and sulfation patterns on individual GAGs. Sulfation along a GAG chain is not uniform and regions of high sulfation, intermediate sulfation, and low sulfation are evident. Sulfation is an important functional determinant and determines the interactive properties of proteoglycans with growth factors, cytokines and morphogens which, regulate tissue development and control cellular behavior in connective tissues during ECM remodeling, tissue repair processes and in tissue morphogenesis. Regions of low and high sulfation are shown in putative HS and CS side chains (a, b). KS has been categorized into N‐linked corneal KSI via asparagine residues (c) and skeletal KSII found in O‐linked attachment to cartilage aggrecan via serine and threonine residues (d, e). Regions along the KS chain labeled 1–5 indicate a multisulfated region (1), monosulfated region (2), non‐sulfated Poly N Acetyl lactosamine stretches (3), the N‐terminal linkage region to asparagine and O‐linked attachment regions to serine and threonine residues in proteoglycan core proteins (4). A specific trifucosylated 3D12/H7 KS epitope that has been identified [122] in the CS1 and CS2 region of cartilage aggrecan and the GAG linkage residues. The 3D12/H7 KS epitope only occurs in cartilage aggrecan suggesting that KSII in cartilaginous tissues may require categorization as KSIIA (d) and KSIIB (e). Modifications along specific regions in CS chains have also been identifiedusing MAbs (f). Diagram of a KS‐I chain showing the epitopes identified by the KS MAbs 5D4 to multisulfated regions and MAb R10G and 1B4 to monosulfated regions (g). The BKS‐1 epitope in KS‐I (h) and KS‐II (i) generated by keratanase pre‐digestion.

**FIGURE 2:**
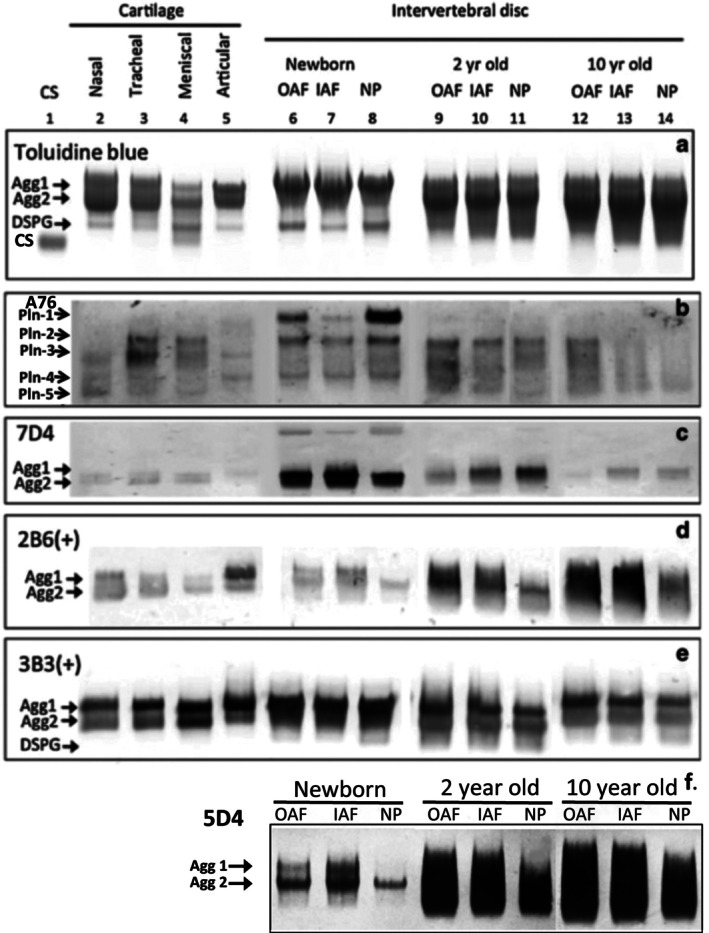
Separation of proteoglycans by native composite polyacrylamide agarose gel electrophoresis and immunoblotting [140] using a range of MAbs to perlecan domain‐1 (b) and to CS (c–e) and KS (f). Several PG populations are discernable by toluidine blue staining (a). Perlecan appears highly susceptible to fragmentation by proteases with five species stained with MAb A76 to perlecan domain‐1 in these tissues (b). MAb 7D4 [123] to a specific CS sulfation motif stains 3 PG populations including perlecan and 2 aggrecan populations (c). MAbs 3B3 and 2B6 to C6S and C4S [124, 125] show widespread distrubutions to two aggrecan populations in all samples (d, e). MAb 5D4 [128] to KS identifies two discrete aggrecan populations in newborn IVD samples however in older IVD specimens KSPGs are significantly more polydisperse (f). Figure reproduced from [140] with permission.

The GAG side chains of PGs are highly heterogeneous; their spatio‐temporal localizations contribute to tissue morphogenesis through variable ligand interactivities [7, 8, 10, 115, 119, 120, 121]. The GAG side chains of IVD PGs equip them with diverse interactive properties with a large range of ligands including growth factors, morphogens and structural ECM glycoproteins. This facilitates the stabilization of tissues, modulation of cellular activity in tissue development and physiological processes that regulate IVD functional properties to maintain optimal IVD mechanics. The sulfation of GAGs is a key functional determinant and varies along an individual GAG chain (Figure 1). Advances in glycomics, analytical methods including sequencing of GAG chains, have facilitated the determination of GAG structure–function inter‐relationships. The development of monoclonal antibodies (MAbs) (Table 2) to specific native chondroitin sulfate (CS), dermatan sulfate (DS), heparan sulfate (HS) and keratan sulfate (KS) sulfation motifs has enabled the identification of high and low sulfation regions along GAG chains [119, 120, 121, 123, 124, 125, 138, 139]. GAG‐depolymerizing enzymes such as chondroitinase AC, B and ABC have greatly aided in the identification of DS, C4S and C6S distributions in GAG chains; however these are not mammalian enzymes and until relatively recently there were no human proteins known with similar enzymatic capabilities. Identification of 3B3(−) and 2B6(−) epitopes in IVD PGs was puzzling until HYAL 4 was shown (despite its misnaming) to actually be a CS hydrolase capable of generating these epitopes [126]. Tissue immunolocalizations carried out using these GAG sulfation motif MAbs in a range of tissues including the IVD, demonstrated they regulate tissue morphogenesis and are associated with progenitor stem cell populations with roles in tissue development and tissue repair processes [119, 120, 121, 123, 124, 125, 138, 139]. Some isolated purified PGs have also been identified with specific CS sulfation motifs (see Figure 2c lanes 6–8) [140, 141].

**TABLE 2:** Monoclonal antibodies developed to proteoglycan glycosaminoglycan determinants.

MAb clone #	PG epitope identified	References
3‐B‐3(+)*	Chondroitin‐6‐sulfate reducing terminal un‐saturated uronate stub epitope on linkage region generated by chondroitinase ABC	[123, 124, 125]
3‐B‐3(−)	Chondroitin‐6‐sulfate non‐reducing terminal un‐saturated stub uronate epitope generated by HYAL4 CS hydrolase	[126]
2‐B‐6(+)*	Chondroitin‐4‐sulfate reducing terminal un‐saturated uronate stub epitope on linkage region generated by chondroitinase ABC	[123, 124, 125]
7‐D‐4	Native specific CS sulfation motif identified using partial digestions with chondroitinase ABC. Sensitive to total chondroitinase ABC digestion	[123, 124, 125]
4C‐3	Native specific CS sulfation motif identified using partial digestions with chondroitinase ABC. Sensitive to total chondroitinase ABC digestion	[123, 124, 125]
5‐D‐4	Hexa sulfated KS octa‐saccharide and a linear dodecasaccharide containing N‐sulfated glucosamine in KS multisulfated regions	[127, 128]
MZ‐15	Hepta and octa‐saccharide KS oligosaccharides in multisulfated KS regions	[127]
IB‐4	Tetrasulfated hexasaccharide in linear KS mono‐sulfated region	[127]
R10G	Low sulfation KS in mono‐sulfated regions	[129, 130, 131]
294‐1B1	Low sulfation KS decorating podocalyxcin	[132]
3D12/H7	Sulfated fucosylated poly‐N‐acetyllactosamine linkage region epitope distributed throughout the CS1 and CS2 region of cartilage aggrecan	[122]
BKS‐1(+)**	KS neo‐epitope, 6‐sulfated GlcNAc adjacent to a nonsulfated lactosamine disaccharide in reducing terminal PG linkage region exposed by keratanase‐1 pre‐digestion.	[10]
F69‐3G10(+)***	HS oligosaccharide containing an unsaturated uronate at the non‐reducing end generated by heparanase‐3	[133]
F58‐10E4	Mixed HS domains containing both *N*‐acetylated and *N*‐sulfated disaccharide units. HS saccharide sequence includes an *N*‐unsubstituted glucosamine	[133, 134, 135]
HepSS‐1	*N*‐Sulfated HS domains	[135, 136]
JM 403	HS saccharide sequence rich in glucuronate and *N*‐unsubstituted glucosamine residues	[134, 135]
NAH46	HS saccharide sequence containing nonsulfated disaccharide units	[137]

*Note:* (+) = *chondroitinase pre‐digestion, **keratanase‐1 pre‐digestion, ***heparanase‐3 pre‐digestion.

A number of monoclonal antibodies to CS sulfation motifs have been developed to identify these specific regions in CS GAG chains [125]. These include MAb 2B6(+) and 3B3(+) which, are used in conjunction with the CS depolymerising enzymes chondroitinase AC and ABC to identify C4S and C6S respectively and MAbs to specific native CS sulfation motifs including MAb 6C3, 7D4 and 4C3 whose epitopes have been identified using graded partial CS digestions with chondroitinase AC/ABC [123]. The 3B3(−) epitope is an unsaturated C6S epitope identified in tissues that does not require chondroitinase ABC pre‐digestion to generate it and should not be confused with the 3B3(+) epitope which, requires chondroitinase pre‐digestion. The mammalian enzyme HYAL4, a CS hydrolase can generate the 3B3(−) epitope in tissue proteoglycans [120] explaining the very specific localisation patterns of the 3B3(−) epitope in tissue morphogenesis [115, 119, 138]. KS chains are typically 5–15 kDa in size, the CS chains in aggrecan are 20 kDa and CS chains in versican are 45 kDa and contain ~20 disaccharides.

## 3 Methods That Have Been Used to Examine the Biodiversity of Native IVD Proteoglycans

Composite agarose‐polyacrylamide gel electrophoresis (CAPAGE) using 1.2% polyacrylamide and 0.6% agarose composite gels is a native electrophoretic system which, separates PG populations primarily on the basis of native charge: mass ratio. The electrophoretic gel used is very soft and has only minimal sieving effects on the separation of very large PGs. This electrophoretic system has proven to be an extremely useful way of examining the complexity of native PGs in cartilaginous tissues and their glycan substructures [140]. Proteoglycans in their fully solvated GAG‐substituted forms are too large to be examined by SDS PAGE where the extremely large size of native PGs prevents their resolution in SDS PAGE gels, necessitating the enzymatic removal of GAG chains prior to electrophoresis to significantly reduce their molecular dimensions. Furthermore, in SDS PAGE, PGs are separated as denatured core protein complexes with SDS and these separate due to the charge properties provided by the SDS component of the protein‐SDS complex. Since GAG side chains have been removed prior to electrophoresis, significant information on the glycanation of PGs is not possible using this technique. CAPAGE does not suffer from this limitation; immunoblotting using many GAG MAbs, have been employed to provide useful information on the GAG diversity of PGs. Furthermore, separated PGs immobilized on nitrocellulose membranes can be digested with GAG depolymerizing enzymes (chondroitinase AC and ABC) to provide information on the distribution of GAGs with specific sulfation motifs on the separated PGs, resolved in CAPAGE as their native forms [140]. Figure 2 illustrates the complexity of PGs in cartilaginous tissues visualized using some of these MAbs. PGs separated by CAPAGE can be stained with cationic dyes such as toluidine blue, alcian blue or the cationic carbocyanine dye “Stains‐All” which, displays some differential staining properties with different GAGs, staining highly anionic GAGs blue while less acidic GAGs are stained pink, Ca2^+^ binding proteins in muscle also stain intensely with Stains‐All and better than with the commonly used protein stain Coomassie R250. In Figure 2a, the PGs were stained with toluidine blue, a good general‐purpose cationic stain for PGs. Alcian blue cationic phthalocyanine dye is a historic mucopolysaccharide stain [142] which, has been used in a critical electrolyte staining method for GAG [143] using variable concentrations of MgCl_2_ and pH conditions to differentiate variably charged GAGs on the basis of their carboxylate contents and offers semi‐quantitation [144]. At pH 2.5 GAG staining with Alcian blue identifies HA and the sulfated GAGs whereas preferential staining of the sulfated GAGs occurs at pH 0.5 to 1.0 [145]. The method has been largely superseded by superior GAG stains such as Stains‐All which, is more sensitive and also differentially stains GAGs in a range of colors [146]. Stains‐All can also be used to stain HA in electrophoresis gels [147]. Staining methods for HA based on the G1 globular domain of aggrecan used as a bio‐affinity probe have also been developed [148]. Antibodies to PG core protein epitopes can also be used on CAPAGE blots to confirm the identity of glycanated PG species of interest. Biotinylated HA has also been developed as a bioaffinity probe to detect PG species capable of interaction with HA [149, 150, 151]. Biotinylated trypsin can also be used as a bioaffinity probe to detect the KPI domains of bikunin [113, 152].

## 4 Proteoglycans Have Major Roles in Spinal Development

**FIGURE 3:**
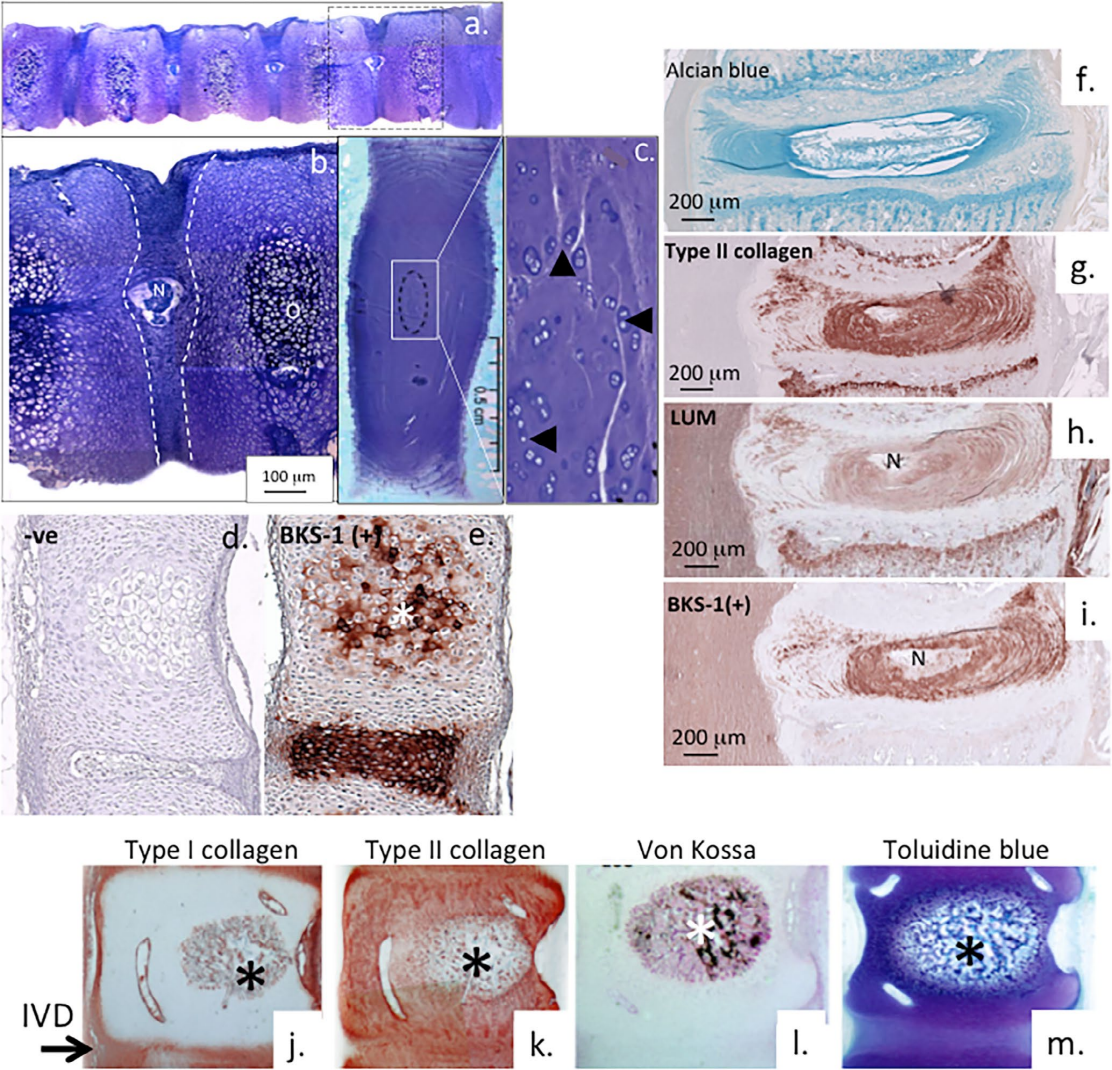
Proteoglycans are major components of spinal tissues during its early development and in the mature IVD. Human fetal thoracolumbar spinal segment (14 weeks gestational age) showing widespread toluidine blue staining of spinal tissue which, is predominantly cartilaginous at this developmental age (a). Notochordal remnants are visible in the center of the developing IVD at 14 weeks, vertebral rudiments are highly cartilaginous at this developmental age (b), hypertrophic central cells in the vertebral rudiment are precursors to development of ossification centers (O). The IVD is better delineated in a 2 year old IVD, the adjacent vertebral body is now bone. A central region of the NP has chondroid progenitor cell clusters (arrowheads) (c). KS localization using the neoepitope antibody BKS‐1 visualizes the IVD and hypertrophic cells in developing ossification centers (O) in the rat E19 lumbar spine (e), negative control (d). Alcian blue staining of GAG in a rat E19 IVD and positive and inferior vertebral growth plates (f), Immunolocalisation of type II collagen (g), lumican (h) and KS using the neo‐epitope KS MAb BKS‐1 using keratanase‐1 to generate the BKS‐1 neoepitope in the KS linkage region (i). Immmunolocalisation of type I collagen (j), type II collagen (k), calcium (l) and GAG using toluidine blue staining (m) delineating ossification centers in the adolescent ovine lumbar spine (f–i). Type I and II collagen show complimentary localizations with the former confined to the periphery of the rudiment while type II collagen is localized in the vertebral rudiment and adjacent IVD. Segments of figure reproduced from [10, 153] with permission. The boxed area in (a) is depicted at higher magnification in (b), the boxed area in (c) is also depicted at higher magnification, groups of chondroid cells are labeled by arrow‐heads. Figure modified from [10] with permission.

Proteoglycans are major tissue components of the human fetal spine, as is clearly shown by toluidine blue staining (Figure 3a,b). The vertebral rudiments are cartilaginous in the fetal human spine. At 14 weeks gestational age hypertrophic cells located centrally in the vertebral rudiment are precursors of the ossification centers that subsequently form in the vertebral rudiment and are part of the tissue morphogentic process that leads to ossification of the vertebral body and development of the spinal column (Figure 3b). Notochordal remnants are also visible in the center of the developing IVDs at this stage of spinal development (Figure 3b). In the adult human spine, the IVDs are more clearly delineated by toluidine blue staining since they are now bordered by ossified vertebral bodies devoid of GAG (Figure 3c). Small chondroid cell groups are also evident in the central NP in a region previously occupied by the notochord [154] (Figure 3c). Isolation of these chondroid cells and culturing in vitro has shown they express several notochordal biomarkers [155]. Single cell transcriptomics identify notchordal marker expression by post‐natal NP cells [156]. Determination of the human notochordal cell transcriptome identifies potential regulators of cellular function in the developing IVD [157]. Use of a KS neo‐epitope antibody BKS‐1(+) has identified prominent localisations in the vertebral rudiment ossification centers and IVD in the E19 rat (Figure 3e). Several KS‐SLRPs have been shown to have roles in bone metabolism including DCN, BGN, CHAD, OMD, PRELP and OGN [158, 159, 160]. Type I collagen was localized predominantly around the periphery, whereas type II collagen was immunolocalised throughout the ossification center and vertebral body rudiment (Figure 3j,k). Von Kossa staining showed Ca2^+^ localisation in the ossification center (Figure 3l).

Alcian blue staining of E19 rat spinal tissues showed GAGs were prominent components of the IVD and VGP and followed a similar distribution to type II collagen which, is also a prominent component of cartilaginous tissues (Figure 3f,g). Immunolocalisation of lumican showed similar distributions in the IVD and vertebral growth plate (VGP) but it was also prominently expressed in the spinal cord (Figure 3h). Use of the KS neo‐epitope antibody BKS‐1 following keratanase‐1 pre‐digestion immunolocalised KS in the IVD and spinal cord but not in the VGP (Figure 3i). Several SLRPs are known to be present in the growth plate including DCN, BGN, FMOD and LUM [161]; however the lack of BKS‐1(+) immunoreactivity in the E19 rat showed the form of lumican found in the VGP was not substituted with KS. Non‐glycanated and glycanated forms of DCN have previously been observed in the human IVD [117]. Non‐glycanated forms of BGN have also been observed in articular cartilage [162]. Non‐glycanated forms of lubricin have also been reported [163]. A reduction in DCN glycanation has been reported in cortical bone from aged wild type mice [164]. The GAG chains of FMOD are also shortened with aging until the non‐glycanated form becomes the predominant form present in aged tissues [165]. N‐terminal glycosylation sites in SLRPs can be post‐translationally modified where they become incapable of acting as a GAG chain attachment site. Thus while SLRPs may have 3–4 glycosylation sites with potential as GAG binding sites, in practice not all of these sites may be occupied.

## 5 Spatiotemporal CS Sulfation Motifs Expressed in Spinal Tissue Morphogenesis

**FIGURE 4:**
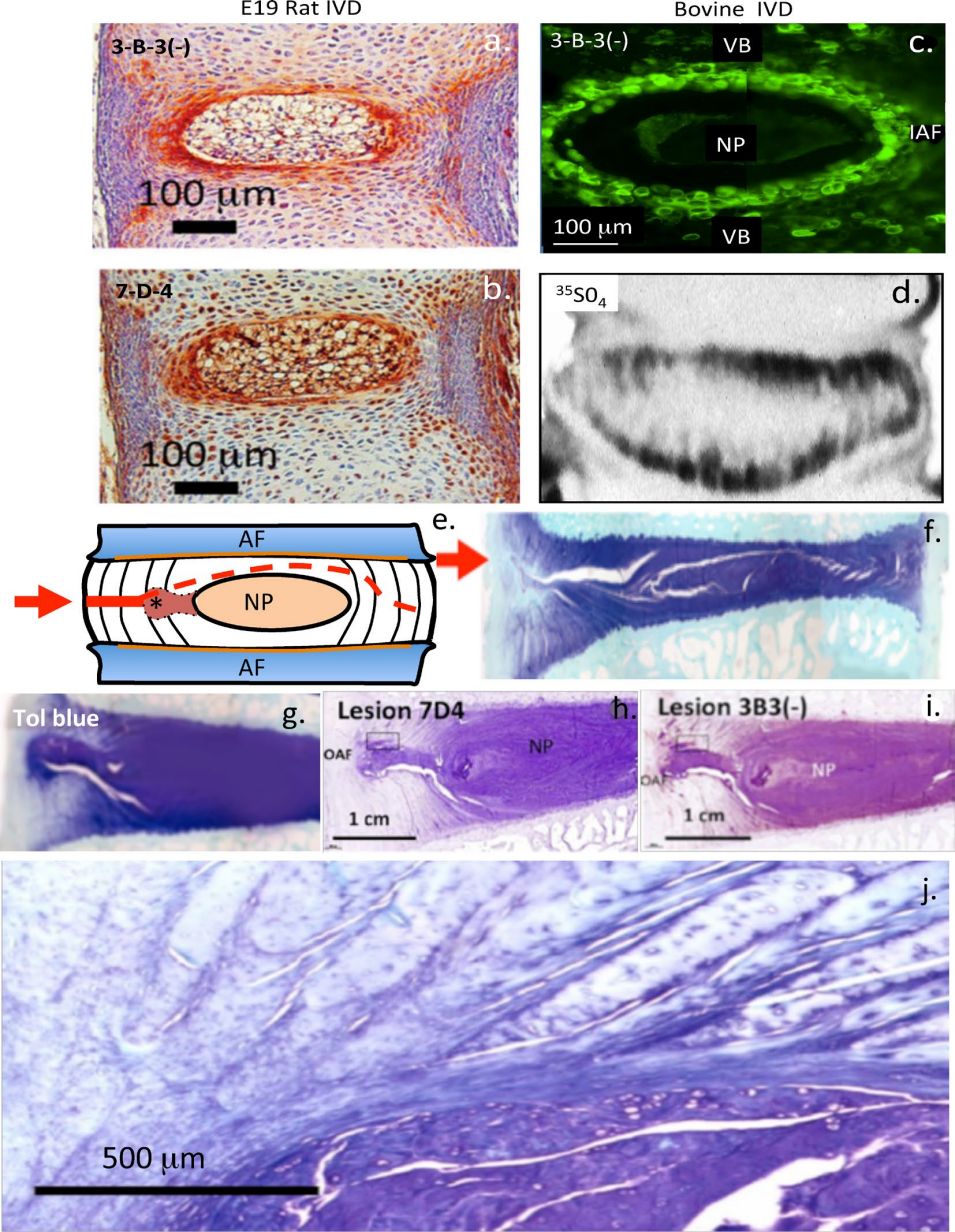
Histological demonstration of the expression of CS sulfation motifs in intervertebral disc development (a–c) in regions of intense metabolic activity (d) and in remodeling IVD tissue eliciting a repair response (g–j) in IVDs containing controlled annular lesions used to induce experimental IVDD.

The 3‐B‐3(−) and 7D‐4 CS sulfation motifs are prominently expressed in the transitional zone between the inner AF and NP in the E19 rat and juvenile bovine IVD and these represent markers of tissue morphogenesis (Figure 4a–c) [138]. The transitional zone is a region of intense metabolic activity as demonstrated by the [35] SO_4_ incorporation into IVD PGs (Figure 4d). This region of the IVD represents a physis or growth plate in IVD development. The 3‐B‐3(−) and 7‐D‐4 CS sulfation motifs are also focally expressed in chondroid outgrowths from the NP which, remodel the inner regions of experimental annular lesions that have been used to induce IVDD [166] (Figure 4h,i). These chondro‐proliferative regions may represent an endogenous repair response by a resident chondroprogenitor cell population (Figure 3c) [121, 154] which, prevents the propagation of the annular lesion deep into the IVD towards the contralateral AF and which, results in IVDD in the absence of this cellular response (Figure 4f) [167]. The 3B3(−) and 7D4 CS sulfation motifs thus also represent markers of resident stem cell activity which, promotes the repair of such annular lesions (Figure 4j) [121, 154].

## 6 Subtle Variations in Aggrecan Structure in Early Spinal Development

Figure 6 shows subtle variations in aggrecan structure in developmental tissues that will form the spinal column and IVD. Neural tube aggrecan contains significantly less KS than cartilage aggrecan (Figure 6f), aggrecan found in the developing notochord may not contain KS (Figure 6g). Furthermore, aggrecan involved in the formation of the neural tube and notochord precursors of the spinal column has some of its CS chains replaced by the human natural killer HNK‐1 trisaccharide (Figure 6f,g). The HNK‐1 epitope has instructive properties over neural crest cells and directs formation of the neural tube and notochord; however this epitope is no longer a component of the aggrecan core protein in mature IVD tissues [168, 169].

## 7 Variable Structure of Aggrecan in Adult Cartilaginous Tissues

**FIGURE 5:**
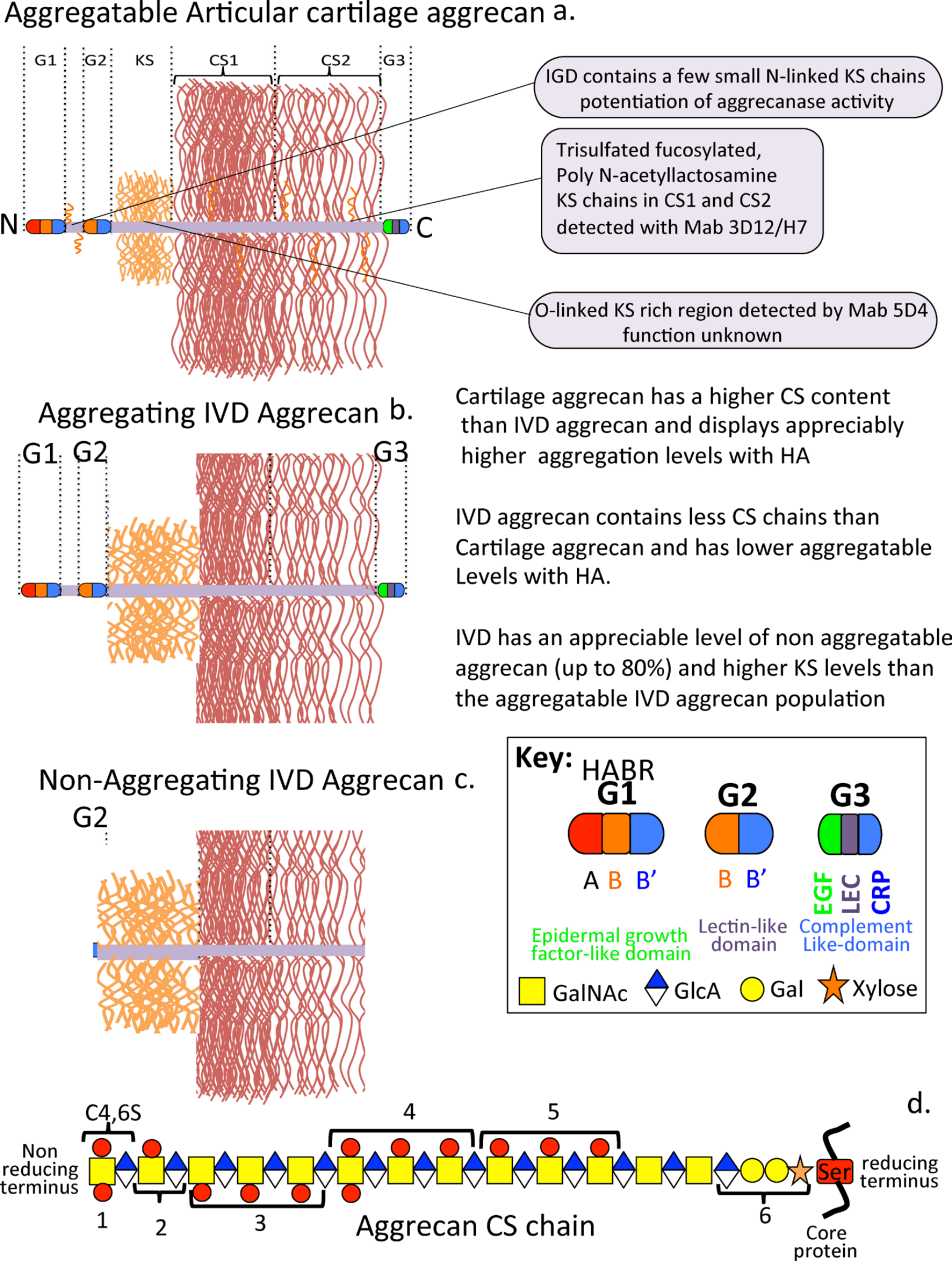
A schematic comparison of aggrecan structural organization in articular cartilage and IVD. For an explanation of the annotated features in the CS chain see Table 3. The variable sulfation along a CS chain is represented by a red circle. The icons shown in d. are standard SFNG glycan icons.

The IVD and articular cartilage are often considered similar tissues which, is not unreasonable given that they are tissues designed to resist compressive loading and they both contain similar ECM components that are assembled to provide a supportive weight‐bearing tissue. However, the spatial organization of these ECM components is quite dissimilar in each of these tissues. Furthermore, aggrecan is also quite dissimilar in composition in these tissues. IVD aggrecan contains significantly more KS than cartilage aggrecan and is concentrated in the NP of the IVD. Aggrecan aggregation levels with HA in cartilage and IVD are also quite dissimilar. Cartilage aggrecan displays high aggregation levels with HA, typically ~80%, while in the IVD aggregation levels may be as low as 30% in the same individual (Figure 5). IVD aggrecan is also significantly more heterogeneous than cartilage aggrecan and contains smaller molecular weight aggrecan species that cannot aggregate with HA reflecting protease degradation of the IVD PGs. Degraded aggrecan in articular cartilage in contrast is lost from the cartilage ECM, diffusing into the synovial fluid, whereas in the IVD these are retained within the IVD. IVD aggrecan also becomes progressively less extractable with advancing age compared to cartilage aggrecan due to G3 mediated interactions with structural glycoproteins and collagen networks which, retain it more tenaciously in the tissue residue even after extraction with 4 M GuHCl (guanidine hydrochloride), a typical chaotropic dissociative extractant commonly used to extract aggrecan from cartilaginous tissues [183].

## 8 Aggrecan CS Side Chain Diversity

Aggrecan contains approximately 8 to 10 KS chains and ~100 CS chains, each GAG is spaced ~1 to 1.5 nm apart and these range in size from 14 to 21 nm in length [184].

Aggrecan is widely distributed in the articular hyaline cartilages of diarthrodial joints, but it also occurs in elastic fibrocartilages of rib, nasal and tracheal cartilages, larynx, outer ear and the epiglottis [185, 186, 187, 188] and is an important functional component of the myocardial ECM [189] in fetal heart development contributing to the resilience of the endocardium, myocardium, epicardium and valve leaflets of mature heart tissue [190, 191]. Aggrecan is also found in the CNS and PNS in the perineuronal net (PNN) in aggrecan‐HA‐tenascin C aggregate structures. These are specialized forms of ECM with roles in neuroprotection and synaptic plasticity [192, 193, 194].

Aggrecan is a major structural proteoglycan of articular and other weight‐bearing tissues with instructive roles over neural crest cells during the formation of the notochord precursor to spinal tissues [168]. Aggrecan from articular cartilage contains KS and CS side chains which constitute ~90% of its mass. CS is the major aggrecan GAG and is organized into CS1 and CS2 regions. A key property of aggrecan is its interaction with HA via its G1 N‐terminal globular domain; this is important in the tissue hydration and osmoregulation [195, 196], and in weight‐bearing in joint cartilages [197] and IVD [15, 198] and hydrates soft tissues such as the brain [199, 200, 201] (Figure 5, Table 3). Several years ago MAbs 3‐B‐3(−) and 7‐D‐4 identified chondrocyte clusters in pathological OA canine and human articular cartilage [202]. At this time, pre‐dating knowledge of tissue stem/progenitor cell niches, these cell clusters were considered a classical feature of the onset of late‐stage degenerative joint disease. An alternative explanation has now emerged; these “chondrocyte‐clusters” may arise from adult stem/progenitor cell niches [203, 204]. The 3‐B‐3(−), 4‐C‐3 and 7‐D‐4 CS sulfation motifs occur in fetal development and are markers of anabolic processes in transitional tissues. An important feature of the stem/progenitor cell niche is the sulfation of GAG side chains; variable expression of GAG sulfotransferases and glycosyl transferases in stem/progenitor cell niches supports this interpretation [205] and the expression of Notch 1 and CD166, biomarkers synonymous with the stem cell niche [121, 180, 206].

**TABLE 3:** Structural/Functional Diversity of the CS side chains of Aggrecan (see Figure 5).

Label	Structural/Functional features of annotated region	References
1	Non‐reducing terminal disulfated CS epitopes interactive with morphogens such as IHH.	[172, 173, 174]
2	New non‐reducing terminus generated by HYAL4 digestion generates 3‐B‐3(−) epitope	[175]
3	Increased C‐6‐S 6C3 levels in overloaded cartilage regions and with aging and reduced levels in OA are due to alterations in the expression of sulfotransferases	[176, 177, 178, 179]
4	Region of CS chain detected by MAb 4‐C‐3, C‐4‐S epitopes predominate in fetal cartilage	[60, 180, 181]
5	Region of CS chain detected by MAb 7‐D‐4	[60]
6	Linkage region to Serine residues on aggrecan core protein and GAG accepter region involved in the biosynthesis of CS chains.	[182]

Approximately 2 in 7 CS chains in aggrecan are terminated in non‐reducing terminal 4,‐disulfated GalNAc; this varies with age and cartilage type, 4 in 7 CS chains are terminated by 4‐sulfated GalNAc, and 1 in 7 by a GlcUA linked to 4‐sulfated GalNAc, 4,6‐disulfated GalNAc residues are 60‐fold more abundant in this location than in interior regions of the CS chain [207]. CS chains terminated in 4‐sulfated GalNAc predominate in aggrecan from fetal to 15‐year‐old knee cartilage; in adults 22 to 72 years old, 50% of the CS chains were terminated in 4,6‐disulfated GalNAc. GlcUA‐4‐sulfated GalNAc disaccharides terminated 7% of CS chains in fetal to15 15‐year‐old cartilage but fell to 3% in adults whereas GlcUA‐6‐sulfated GalNAc represented 9% of the CS chains in fetal to 72‐year‐olds. This disaccharide is recognized by MAb 3‐B‐3(−) [172]. The distribution of 4‐ and 6‐sulfated CS epitopes is variable along an aggrecan CS chain and is influenced by the age of the cartilage and its weight‐bearing history [208]. C‐4‐S is more predominant in aggrecan from fetal and juvenile articular cartilage and occupies a central region in the CS chain; non‐sulfated chondroitin is more predominant towards the linkage region. C‐6‐S has a predominant distribution towards the non‐reducing terminus and is more abundant in mature cartilage [60, 208] (Figure 5d). Graded partial digestions of CS chains with chondroitinase ABC have revealed regions along the CS chain where MAbs 6C3, 4C3 and 7D4 are immunoreactive [60]. MAb 6C3 reacts optimally with CS chains in the non‐reducing terminus region; this reactivity is removed by chondroitinase digestion.

Further digestion of the CS chain removes MAb 4C3 reactivity and continued digestion removes reactivity to MAb 7‐D‐4 providing information on the relative placement of these sulfation motifs along the CS chain, The fine structure of these CS sulfation epitopes has yet to be identified. MAb 3‐B‐3 identifies a non‐reducing terminal GlcUA‐GalNAc‐6‐sulfate disaccharide 3‐B‐3(−) epitope; the 3‐B‐3(+) stub epitope attached to the linkage region is also generated by chondroitinase ABC [60].

## 9 The Structural Organization and Function of IVD Proteoglycans

### 9.1 Asporin

Asporin (periodontal ligament‐associated protein 1 [PLAP1]) levels are elevated in IVDD particularly in the outer AF, and it has been suggested as an early marker of IVDD [11, 12, 13, 209]. In NP cells, IL‐1β increases asporin expression via the NF‐κB p65 pathway during IVDD [12].

### 9.2 Aggrecan and Versican

Aggrecan (ACAN) and versican (VCAN) are related large CS PGs that share G1 and G3 globular domains at their N‐ and C‐termini; however they have differing compositions and structural organization [210]. Their N‐terminal G1 domains bind HA forming link protein‐stabilized ternary complexes with space‐filling and hydrating properties through the formation of an HA‐rich matrix, that regulates HA‐mediated cell signaling [115, 211, 212]. The C‐terminal G3 domain of ACAN and VCAN binds ECM components, forming supramolecular structures that store transforming growth factor β (TGFβ) and bone morphogenetic proteins (BMPs) and have roles in their regulation and the cell signaling processes they promote [211]. EGF‐like motifs in the G3 domain have been proposed to act directly like an EGF ligand directing cell migration, cell proliferation and differentiation in tissue development and ECM remodeling in tissue repair processes.

**FIGURE 6:**
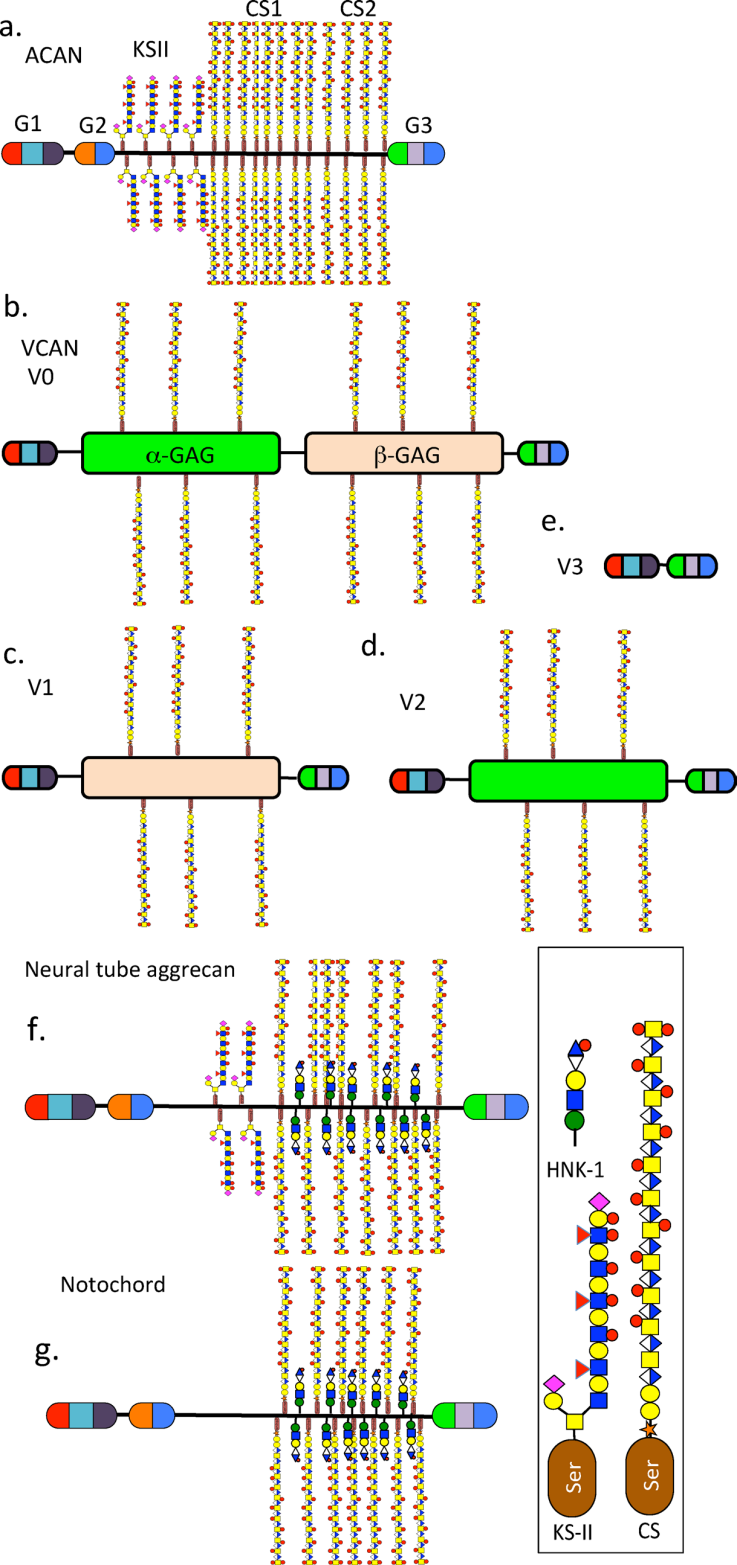
Schematic depictions of the structural organization of aggrecan (a) and the Vo (b), V1 (c), V2 (d) and V3 isoforms of versican (e) showing their globular G1, G2, and G3 domains KS and CS rich regions of aggrecan and the GAG‐α and GAG‐β subdomains of versican. The G1 domain consists of IgA and proteoglycan tandem repeat B, B′ folds which, bind HA; the function of the G2 domain in aggrecan is unknown. The G3 C‐terminal domain of aggrecan and versican consists of EGF, lectin‐like and Complement CRP subdomains, which interact with cells and ECM components forming extended aggrecan and versican collagen networks with apparent biomechanical biosensory functions that deliver ECM signals to direct cellular behavior and regulate tissue function and homeostasis. Subtle variations in aggrecan structural organization showing the reduced KS‐rich region in neural tube aggrecan (f), replacement of CS chains by HNK‐1 trisaccharide and absence of KS chains in notochordal aggrecan (g) [168, 169, 170, 171].

The KS chains in articular cartilage aggrecan are relatively small (5–15 kDa), mainly grouped in a KS‐rich region adjacent to the G1 and G2 globular domains. Isolated fucosylated KS chains have also been uniquely detected in the CS1 and CS2 regions of the C‐terminal half of human cartilage aggrecan [122] (see Figure 1d). KS has unique functional capability; however the function of KS in aggrecan has yet to be fully determined [139]. Not all forms of aggrecan contain KS chains suggesting they may be some form of structural specialization in some tissue contexts [139]. The aggrecan core protein is 200–250 kDa in size [212], versican has an appreciably larger core protein of ~360 kDa but lacks the G2 and KS chains of aggrecan. Versican has 12–15 CS chains compared to ~100 CS chains in aggrecan. The CS chains in versican are larger than the CS chains in aggrecan (45 vs. 20 kDa). Versican has GAG‐α and GAG‐β domains which, occur in four alternatively spliced forms (V0, V1, V2 and V3) (Figure 6b–e). These variations in structure have specific roles in tissue development including the IVD [34, 115] affecting cellular migration and proliferation in response to interactions with growth factors and cytokines [211, 213, 214].

Versican G3 domain contains EGF motifs that enhance cell proliferation, adhesion, migration and roles in IVD development [21, 215]. Versican also interacts with inflammatory cells via HA, CD44, P‐selectin glycoprotein ligand‐1 (PSGL‐1), and TLRs to induce inflammatory cytokines such as TNFα, IL‐6, and NFκB [216]. Versican's interactions with growth factors and cytokines also regulate inflammatory processes providing tissue protection. Versican also interacts with a number of ECM structural proteins and regulates ECM structural organization [211, 213].

### 9.3 Perlecan in the Disc

**FIGURE 7:**
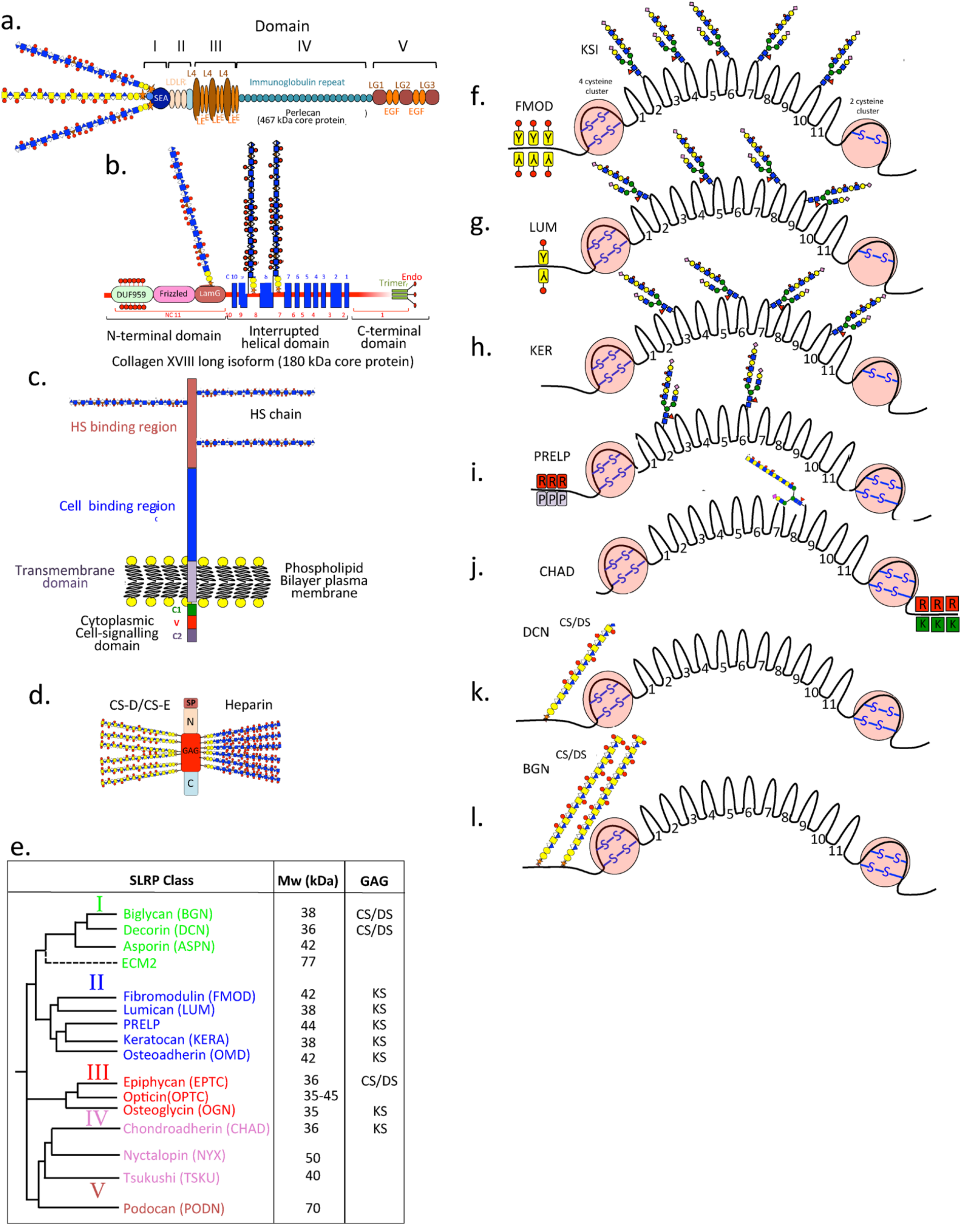
Schematic depiction of the structural organization of HSPGs identified in the IVD. Perlecan (a). Collagen XVIII occurs in 3 alternatively spliced isoforms (b). The structure depicted is the long 180 kDa isoform which, has a heavily glycosylated N‐terminal DUF959 domain of unknown function and a Frizzled domain which, when proteolytically released can inhibit Wnt/β‐catenin signaling. The central region of collagen XVIII contains 10 interrupted collagenous domains with intervening non‐collagenous domains and 2 attached HS chains. The C‐terminal trimeric domain contains an anti‐angiogenic module called endorepellin (b). Syndecan‐4 contains 3 HS chains attached to its C‐terminal HS binding region (c). Serglycin is a small 14 kDa core protein intracellular PG which, forms inactive cytoplasmic granule complexes with serine proteinases and a range of bioactive proteins which, control inflammation, wound repair and cellular invasion. The central region of the Serglycin core protein is its GAG attachment region (d). Dendrogram and classification of the small leucine repeat proteoglycans showing how inter‐related these proteins are to one another and categorization of the SLRPs into five classes (e). Schematic depictions of KS and CS/DS IVD SLRPs showing the central leucine rich repeat domains and flanking N and C‐terminal disulfide stabilized globular domains (f–l). In FMOD and LUM sulfated tyrosine residues are present in the N‐terminus (f, g). PRELP has characteristic Arg and Pro residues grouped in the N terminus while CHAD has Arg and Lys residues grouped in the C‐terminal extension (i, j). Decorin (k) has a single CS or DS chain attached to the N‐terminus while biglycan has two GAG chains (l).

Perlecan is a five‐domain modular multifunctional large IVD proteoglycan (Figure 7a) which, promotes chondrogenesis, spinal development, tissue stabilization, tissue repair and provides cell‐matrix communication and sensory and osmoregulatory properties to disc cells which, aids in optimal tissue function and the maintenance of tissue homeostasis [26, 27, 28, 29]. Domain 1 of perlecan has 3 GAG chains containing two attached HS chains and one CS chain; domain V also has a single GAG chain which, may be a CS or HS chain or may be unoccupied. The perlecan HS chains sequester growth factors, presenting these to and activating cognate receptors to promote cellular proliferation and differentiation and regulate chondrogenesis and spinal development [32, 217, 218, 219]. Domain II of perlecan is a VLDL‐like receptor which, binds morphogens such as Wnt and Shh. These are poorly soluble proteins; thus perlecan aids in their transportation and the formation of morphogen gradients that drive tissue development. Perlecan domain III binds FGF‐7 and 18 and has structural and cell adhesive roles [220]. Perlecan domain IV, an immunoglobulin repeat domain, has cell attachment and matrix stabilizing properties and self‐aggregative properties of perlecan [221]. Perlecan domain V, contains three laminin type G (LamG) and four epidermal‐growth‐factor‐like (EGF) modules that promote tissue repair through interactions with VEGF, VEGF‐R2, α2β1 integrin and α‐dystroglycan [222, 223, 224].

### 9.4 Collagen XVIII in Disc

**FIGURE 8:**
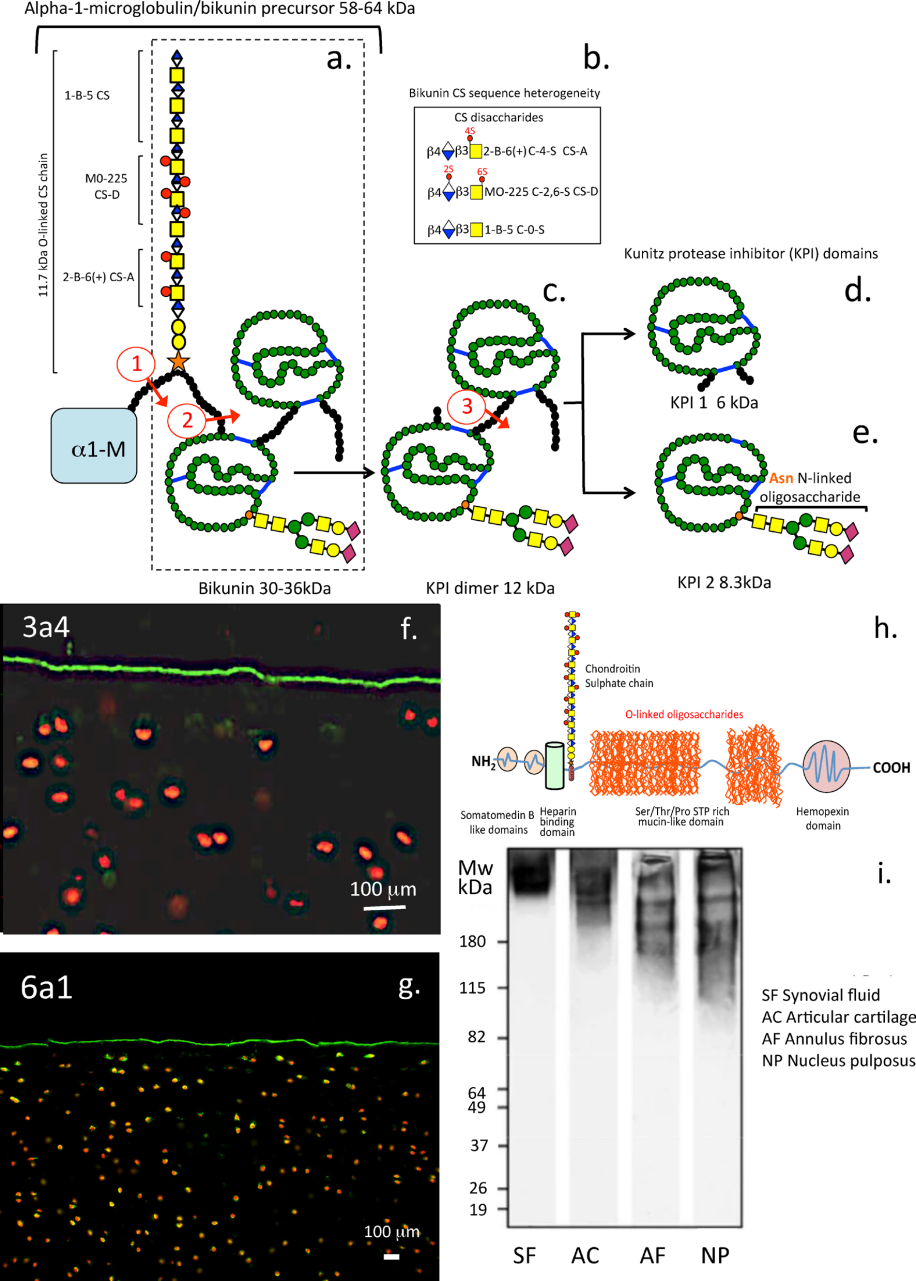
Schematic representation of bikunin the light chain component of ILI. Bikunin occurs as a precursor attached to α1‐microglobulin. Proteolytic processing (1) results in the release of bikunin (a). Bikunin contains a 7 kDa CS side chain containing CS‐A, CS‐D and non‐sulfated chondroitin (b) and two Kunitz protease inhibitor (KPI) domains (c–e). KPI‐2 contains an N‐linked oligosaccharide attached through asparagine (e). Proteolytic processing releases the KPI dimer and the KPI‐1 and KPI‐2 domains (2, 3). The KPI domains have potent inhibitory activity against leucocyte elastase, cathepsin G, trypsin, chymotrypsin, and kallikrein. Immunolocalisation of lubricin to the cell surface of articular cartilage using MAb anti‐lubricin antibodies 3a4 and 6a1 (f, g). Lubricin is also synthesized by chondrocytes and synoviocytes in joint tissues and by IVD cells. Lubricin has one CS chain, but may also be synthesized as a glycoprotein form devoid of GAG. Lubricin has extensive glycosylation through its mucin‐like domains (h). Lubricin is a high molecular weight PG in synovial fluid and in articular cartilage while it is fragmented in IVD tissues (i). Segments a‐e modified from [113], segments (f–i) modified from [229].

Altered expression of minor collagens is a marker of early IVDD; these include collagen VI [225], collagen IX (COL9) [226], collagen X [227], collagen XI, collagen XII [225], collagen XV [225], and collagen XVIII [225] these affect NP and AF structure and accelerate disc degeneration. A spectrum of diseases is caused by > 1000 collagen mutations in 12 collagen types including collagen XVIII [38]. These diseases include osteogenesis imperfecta, many chondrodysplasias, subtypes of Ehlers‐Danlos syndrome, Alport syndrome, Bethlem myopathy, subtypes of epidermolysis bullosa, Knobloch syndrome, some forms of osteoporosis, skull malformations, arterial aneurysms, OA and IVDD [36, 37, 38]. Mutations in the collagen XVIII gene are associated with retinal abnormalities; collagen XVIII is critical for normal blood vessel formation in the eye [228]. Collagen XVIII mutations also affect neural tube closure during spinal cord and brain development resulting in abnormalities in brain structure and function in adults. Collagen XVIII is a HS PG multiplexin, that occurs as 3 alternatively spliced forms containing multiple triple‐helical collagenous domains, interrupted by non‐collagenous domains [36, 37]. A long isoform of collagen XVIII has an N‐terminal domain homologous to the extracellular portion of frizzled receptors which, can inhibit Wnt/β‐catenin signaling after its proteolytic release; this domain is not expressed in the “short” isoform of collagen XVIII (Figure 8b). Multi‐site proteolytic processing in the C‐terminal domain results in the release of endostatin, a potent antiangiogenic peptide module with anti‐tumor activity. Mature type XVIII collagen interacts with laminin, perlecan, nidogen, and fibulins forming complex tissue‐stabilizing networks. Collagen XVIII is susceptible to cleavage by MMP‐2, ‐3, ‐7, ‐9, ‐12, ‐13, ‐20, MT1‐MMP, and cathepsins releasing 20–30 kDa C‐terminal endostatin fragments [230, 231, 232]. A C‐terminal fragment of endostatin, inhibits TGF‐β induced collagen synthesis [233]. Endostatin signals through a wide range of receptors including integrins αvβ3, αvβ5, α5β1, nucleolin, VEGFR‐1‐3, as well as GPC1‐4 receptors antagonizing the binding of VEGF to the VEGF‐1 and ‐2 receptors and inhibiting the phosphorylation of ERK, p38, Akt, p125FAK, and MAP kinases as part of its anti‐angiogenic and anti‐tumor properties [233].

### 9.5 SDC4

Syndecan‐4 (SDC4) is a transmembrane IVD HSPG (Figure 8c) that modulates interactions with ECM components, growth factors and morphogens through HS‐mediated interactions by its side chains [47, 234, 235]. During tissue repair, cells involved in wound healing have upregulated SDC4 and integrin expression. SDC4 regulates wound repair through activation of protein kinase C [236], focal adhesion kinase [43], and RhoA [237]. SDC4 levels are also elevated in OA chondrocytes and correlated with OA progression [44]. In the hypoxic environment of the healthy IVD, SDC4 plays an important regulatory role in tissue homeostasis [238]. SDC4 has diverse roles in cartilage biology. The SDC ectodomain is shed from the cell surface by a range of proteases but retains its binding properties [239] and can modulate growth factor activity at wound sites [240] promoting tissue repair processes. SDC4 is linked with ADAMTS‐5 activation and cartilage breakdown in OA [44] and has been suggested to be a key therapeutic target in the prevention of IVDD by suppression of JNK/P53 cell signaling [241]. TNF‐α and IL‐1β promote the expression of ADAMTS‐5 and degradation of aggrecan [50] while TNF‐α and TGF‐β1 regulate Syndecan‐4 expression in NP cells [49]. SDC4 also has roles in the formation of the collagenous lamellar structure in the AF [41].

### 9.6 GPC6

The glypicans (GPCs) comprise a family of growth and cell division regulatory PGs. GPC6 is a putative cell surface co‐receptor for growth factors, ECM proteins, proteases and anti‐proteases. Mutations in GPC6 are associated with omodysplasia 1(OMOD1), a rare autosomal recessive skeletal dysplasia characterized by severe congenital micromelia (*reduced head and trunk size and gross skeletal abnormalities*) and shortening of the humerus and femur. OMOD1‐affected individuals have growth plate defects in the distal humerus and complex deformities of the elbow. GPC6 polymorphisms are also associated with lumbar disk herniation [51, 52]. Dysfunctional PGs such as GPC6 represent a functional link between IVDD and skeletal dysplasia [53].

### 9.7 Serglycin

Serglycin is a small molecular weight (17.6 kDa core protein), intracellular heparin‐proteoglycan in some cell types including disc cells, but can also be secreted and incorporated into the ECM [97, 98]. The Serglycin core protein has a central 16 amino acid region which, is substituted with long GAG chains (Figure 7d). Serglycin is expressed by cells of hematopoietic origin including neutrophils, lymphocytes, monocytes, macrophages, platelets, megakaryocytes, and mast cells [97, 99, 100, 242, 243] and by endothelial and embryonic stem cells [98, 99]. Intervertebral disc cells and chondrocytes also express Serglycin [8, 101, 102], IL‐1β or TNF‐α increase serglycin expression in vitro. Serglycin levels are also elevated under inflammatory conditions during cartilage and IVD degeneration [8, 101, 102]. Heparin exclusively substitutes serglycin in connective tissue mast cells [103] however mucosal mast cells, activated monocytes and macrophages contain serglycin substituted with highly sulphated chondroitin‐4,6,‐disulfate; quiescent monocytes contain serglycin substituted with chondroitin‐4‐sulfate [97].

### 9.8 The SLRPs

The SLRPs have been categorized into five families (Figure 7e), Several members are present in the IVD (Table 1) [244]. SLRPs have distinctive boomerang‐type shapes with a convex surface containing attached GAG side chains while the concave surface is available for interaction with fibrillar collagens (Figure 7f–j). The SLRPs have characteristic multiple leucine‐rich repeat (LRR) modules located centrally in the molecule which, are highly interactive with a range of ECM components [245]. Interactions of SLRP members with type I and II collagen regulate collagen fibrillogenesis and have roles in ECM stabilization and in its assembly [92, 246]. The SLRPs have cell‐instructive properties that control ECM assembly processes during development and ECM remodeling in tissue repair and also regulate tissue homeostasis [247, 248].

### 9.9 Lubricin

Lubricin has been detected in the human and canine IVD [108, 249, 250]. The roles of lubricin have been examined in a murine lubricin KO model [250]. Localization of lubricin in tissue areas that experience elevated shear and tensile stresses suggests roles for lubricin in the lubrication of collagen fiber bundles in the knee joint meniscus, the tendon fascicles in digital flexor tendons, and the rotator cuff [249, 251, 252]. This suggests lubricin may have similar roles to play in the lubrication of collagen fibril bundles in the AF subjected to torsional loading. Lubricin also has emerging roles in cellular regulation and in the control of inflammation [111] however the precise role of lubricin in IVD tissues remains to be established. Lubricin contains a single CS chain; however non‐glycanated forms have also been described (Figure 8h) [163]. Lubricin in the AF and NP is more heterogeneous than lubricin isolated from articular cartilage or synovial fluid (Figure 8i).

### 9.10 ITI/Bikunin

The human, canine and ovine IVD contain 120 and 250 kDa ITI‐like serine protease inhibitors (SPIs) active against human leucocyte elastase (HLE), cathepsin‐G, chymotrypsin and trypsin, urokinase, plasmin, kallikrein. ITI is a glycoprotein composed of three polypeptides: two heavy chains (HC1 and HC2) and one light chain (bikunin) (Figure 8a). Bikunin confers the protease‐inhibitor function of ITI while the HC1 and HC2 chains are linked to HA stabilization [5, 253]. ITI has free‐radical scavenging properties and protects HA from depolymerization to a pro‐inflammatory form [254]. Bikunin is the simplest PG, which, is synthesized as a 58 kDa α1‐microglobulin‐bikunin precursor which, is cleaved to release α1‐microglobulin and bikunin. Bikunin is glycosylated at Ser‐10 with a single heterogeneous O‐linked 15–25 kDa C4S chain containing 23–55 saccharide residues; a biantennary N‐linked glycan is also attached at Asn‐45. The 16 kDa bikunin core protein is organized into two Kunitz protease inhibitor (KPI) domains which, are each stabilized by three internal disulfide bonds (Figure 8c). The CS chain of bikunin is variable in length and sulfation containing unsulfated, monosulfated, and disulfated CS‐D disaccharides [255, 256, 257]. The CS chain of bikunin is essential for the transfer of ITI HC chains to HA via a transesterification reaction catalyzed by TSG‐6 [258]. Bikunin/ITI has been immunolocalized throughout the ECM of human IVDs [9], canine IVD tissue extracts contain IαI of ~120 kDa [259]. A retrospective study has shown that small molecular weight serine protease inhibitor fractions of ovine cartilage were in fact the KPI domains of bikunin (Figure 8d,e) [113]. ITI has a diverse range of properties beyond that of protease inhibition including transport of ITI HC chains to HA, anti‐bacterial, anti‐viral, anti‐tumor and anti‐inflammatory innate immunomodulatory properties in host‐defense and cell regulatory processes which, promote wound repair [5]. KPI domain proteins are bi‐functional in several marine, parasite, snake, scorpion and tick venom proteins providing voltage‐gated ion blocking properties and potential clues as to the diversified functional capability of ITI peptide domains in mammalian tissues [5, 260, 261, 262].

## 10 IVD Proteoglycan Fragmentation: Generation of Matricryptin Modules During the Normal Turnover of IVD Tissues and by Catabolic Events

Versican is a dynamic regulator of the ECM [211] and is proteolytically cleaved by ADAMTS‐type proteases in a highly regulated manner [263]. A cleavage product generated by proteolysis of the Glu^441^‐Ala^442^ bond within the VCAN V1 isoform, has been termed versikine [264, 265]. Versikine is a novel bioactive damage‐associated molecular pattern (DAMP) that may facilitate immune sensing of myeloma tumors and modulate tolerogenic consequences of intact VCAN accumulation [265]. Therapeutic versikine administration may potentiate T‐cell‐activating immunotherapies. Precise roles for versikine in the IVD have yet to be defined.

Perlecan is highly susceptible to cleavage by a number of proteases; domain V acts as a vascular PG in its own right. Domain V and its LG3 motif have tissue repair properties, while an anti‐angiogenic peptide termed endorepellin in domain V has anti‐tumor activity through its ability to inhibit angiogenesis and the nutrient supply to tumors [26, 219, 223, 266, 267, 268]. Biglycan is more extensively fragmented in the IVD than other cartilages; some of these fragments may be useful biomarkers of IVD catabolism (Table 3). Fragmentation of BGN (and FMOD) is extensive in regions of annular lesions in an experimental model of IVDD but not in the morphologically normal contralateral AF [78]. A 2 kDa fragment of BGN termed Peniel‐2000 has been shown to be a TGF‐β1 inhibitor and inhibits IVDD [269]. BGN fragments have been correlated with spatio‐temporal remodeling of the AF in an ovine model of IVDD [78] and degenerate human IVDs [60] but also occurs in a range of degenerate cartilaginous tissues [61]. The 9th LRR domain of LUM, lumcorin has been shown to be an inhibitor of MMP‐14 and has anti‐tumor activity, has anti‐angiogenic properties and interacts with α2β1 integrin to inhibit micro‐capillary tube formation [270, 271, 272]. LUM also has a C‐terminal growth factor‐like module called lumikine which, promotes cellular proliferation. The discovery of the C‐terminal peptide anti‐angiogenic module endostatin in collagen XVIII was met with considerable excitement and enthusiasm for its potential use as an anti‐tumor agent through its ability to inhibit the microcapillaries that supply nutrients to tumors. However clinical trials with endostatin have largely failed to demonstrate the utility of endostatin for such applications [273, 274]. A 31–45 kDa fragment of DCN discovered in skin called decorunt shows potential roles in the regulation of collagen networks. DCN is also fragmented in the degenerate IVD and similarly sized fragments to decorunt are also present [60, 275, 276, 277]. Fragments of other members of the SLRPs are also present in degenerate IVDs including BGN, FMOD, LUM and KERA [60]. However, of these only FMOD and BGN fragments have been correlated with spatiotemporal remodeling of the AF and shown to be confined to the annular lesion site but not to the contralateral AF which, was undamaged in an ovine model of IVDD [78]. FMOD is also heavily fragmented in the degenerate human IVD, particularly in scoliotic IVD samples; however appropriate consistent spatial sampling of this tissue is difficult and undoubtedly accounts for the variability in the levels of the fragments observed [60].

## 11 In Vitro Generation of SLRP Fragments Using MMP‐13 and rhADAMTS‐4, 5 Digestion of Normal Articular Cartilage as a Substrate: Comparison With Fragments Seen in Degenerate Cartilages

**FIGURE 9:**
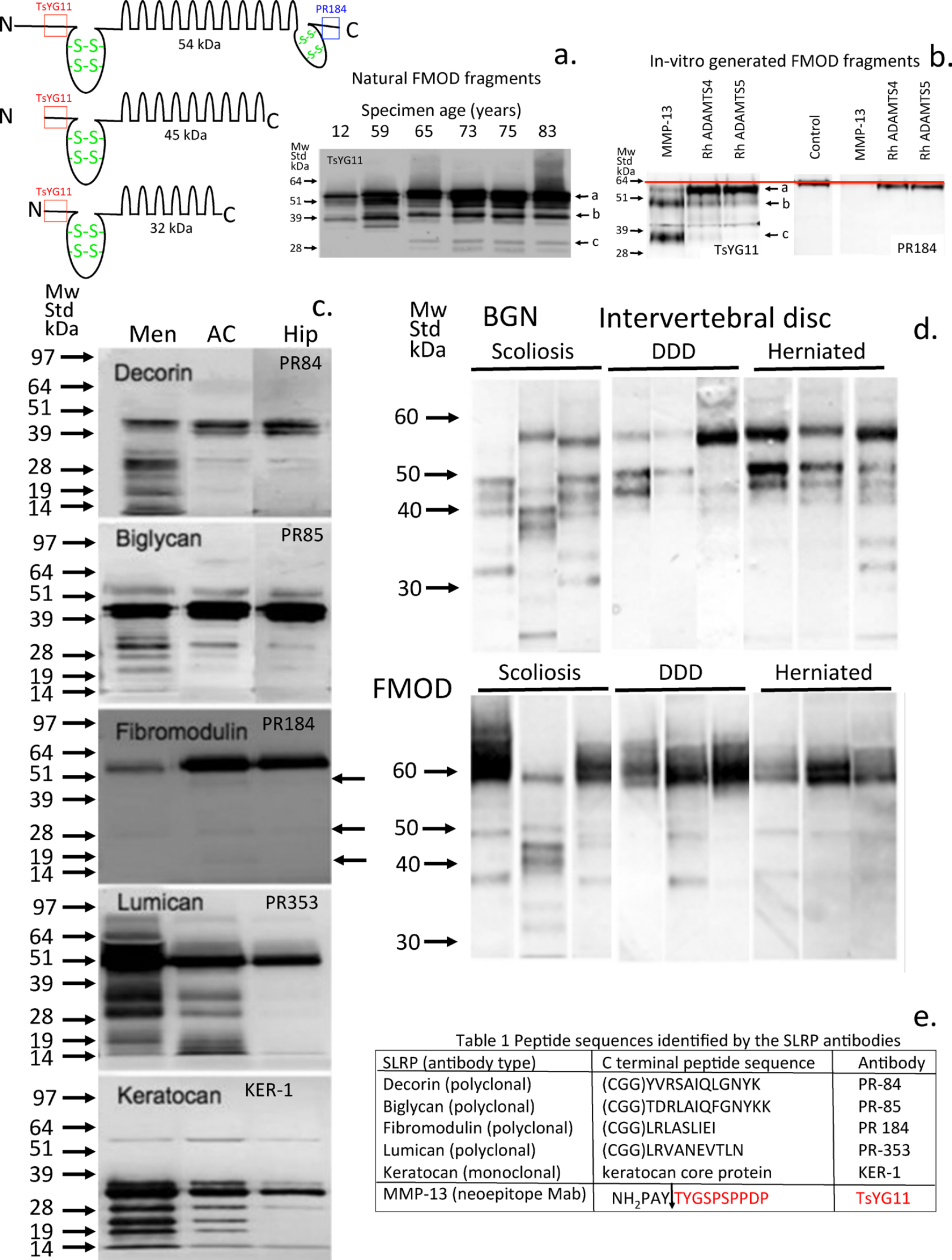
Fragmentation of SLRPs in degenerate cartilaginous connective tissues including meniscus, knee and hip articular cartilage and IVD. Controlled in vitro digestions of non‐degenerate knee cartilage used as a substrate by MMP‐13, ADAMTS4 and ADAMTS5 to generate defined FMOD fragments (a, b) and naturally occurring SLRP fragments in degenerate cartilaginous tissues including meniscus, knee and hip articular cartilage (c) and intervertebral disc (d). Details of the SLRP antibodies used in immunoblotting (e). Figure constructed from data generated in [60, 61, 278]. Figure reproduced with permission.

Full depth human articular cartilage harvested from non‐fibrillated morphologically normal tibial articular cartilage digested in vitro with MMP‐13 and rhADAMTS‐4, 5 has generated some of the naturally occurring SLRP fragments observed in degenerate cartilaginous tissues [278] (Figure 9). Further work may well establish whether these fragments will be useful as specific biomarkers of catabolic processes in the degenerate IVD (see Table 4).

**TABLE 4:** Matricryptic fragments of intervertebral disc proteoglycans.

Proteoglycan	Matrycryptic fragment	Matricryptin features and functions	References
Versican	VERSIKINE	N‐terminal fragment of versican V1 isoform released by ADAMTSs. Has immune regulatory functions however its precise roles in the IVD have yet to be determined	[211, 263, 264, 265]
Perlecan	PERLECAN DOMAIN 5 LG3 in domain 5 Endorepellin module	Promotes tissue repair Promotes tissue repair C‐terminal anti‐angiogenesis peptide module	[26, 219, 223, 267, 268]
Biglycan	PENIEL 2000	En‐silico designed 2 kDa TGF‐β1 inhibitor module, inhibits disc degeneration in animal models of IVDD. BGN fragments are found in degenerate IVDs that correlate with catabolic processes suggesting these may be useful as catabolic biomarkers.	[60, 78, 269]
Lumican	LUMIKINE LUMCORIN	Lumikine, C‐terminal growth factor modulatory peptide, promotes cellular proliferation. 9th LRR module, MMP‐14 inhibitor, inhibits cell migration, anti‐angionic, interacts with α2β1 integrin to inhibit micro‐capillary tube formation, inhibits tumor growth	[270, 271, 272]
Collagen XVIII	ENDOSTATIN	20 kDa C‐terminal anti‐angiogenic peptide module, potential anti‐tumor activity	[273, 274]
Decorin	DECORUNT	Decorunt is a 31–45 kDa fragment of DCN found in skin consisting of the amino‐terminal 40% of decorin with a C‐terminal amino acid sequence of VRKVTF [136]. Decorin is fragmented in the degenerate IVD and similar sized fragments to decorunt are also present. Further work may well establish this as a specific biomarker of catabolic processes in the degenerate IVD.	[60, 275, 276, 277]
Fibromodulin	54, 45, and 32 kDa FMOD fragments	FMOD is extensively fragmented in IVDD 54, 45, and 32 kDa fragments are prominent and can be generated in vitro using MMP‐13 and ADAMTS4 and ADAMTS‐5 to a lesser extent. These fragments show potential as catabolic biomarkers of IVDD and have been spatiotemporally correlated with annular remodeling in an ovine model of IVDD but are not present in the morphologically normal contralateral AF.	[60, 78, 278]

## 12 Future Research on IVD PGs–Some Important Unanswered Questions

Despite the expanding literature on the structure and function of PGs, there are still many questions that need to be answered, for example, (i) Despite the extensive literature on aggrecan over the last two decades, the role of its G2 domain is still unknown, (ii) despite the unique functional capability of KS [139] its roles in aggrecan still need to be clarified, (iii) how does the low aggregatability of IVD aggrecan with HA equate with its attributed roles in weight bearing; in contrast, cartilage aggrecan has high aggregatability in the same individual (iv) neurons live in a carefully controlled ionic microenvironment in the CNS/PNS provided by HA and a number of brain PGs which, establish niche and micro‐gradient ion fluxes that regulate neuronal activation and neurotransmission [200, 201]. How well does the degenerate IVD provide these demanding microenvironmental conditions which, control neuronal viability and function? (v) The degenerate IVD is still a weight‐bearing structure; how does this affect the activity of neuronal ingrowths into degenerate IVDs? While neurons live in a nonweight‐bearing environment in the CNS/PNS they nevertheless are regulated by the mechanosensitive genes Hippo‐TAZ‐YAP system. This may explain the mechanosensitization of nociceptive neurons in degenerate IVDs [279]. (vi) Synthetic biomimetic PGs offer considerable promise in tissue engineering applications [280, 281, 282]. Can synthetic PGs be used in an orthobiological approach to replenish functional components in the degenerate IVD and re‐establish it as an efficient weight‐bearing structure? [26, 283], PGs are important IVD regulatory functional proteins; if some of the above questions can be answered this may not only aid in a greater understanding of the fascinating roles of IVD PGs, but may also uncover potential methods for the alleviation of pain generated by degenerate IVDs and repair of the degenerate IVD.

## 13 Conclusions

There is no doubt that the IVD is a tissue of considerable complexity and functional importance as a weight‐bearing structure. PGs in the IVD are central to the normal biomechanical performance of the composite IVD. IVDD is the number one musculoskeletal condition in terms of socio‐economic impact, years lived with disability, generation of debilitating pain and impairment in the quality of life. PG dysfunction in IVDD provides a number of potential therapeutic targets in this complex multifactorial disease. The glycanation signatures of IVD PGs are pre‐eminent functional determinants over cell regulatory processes in the IVD in health and disease. Significant recent advances in glycomics and glycan analytical techniques are now aiding in the deciphering of how glycans contribute to normal function and degenerative processes in the IVD. Nevertheless, the multifactorial complexity of IVDD remains a significant obstacle to understanding this complex disease process. The recent emergence of new multifunctional IVD PGs such as perlecan, lubricin and bikunin as outlined in this review and biomimetic PGs [282] opens up novel areas of investigation that may well make significant impacts on the elucidation of IVDD degenerative processes and development of new therapeutic approaches in the alleviation of nociceptive pain. Full characterization of the roles of these PGs in IVD pathobiology is thus warranted and these offer exciting possibilities.

## Funding

This study was funded by The Melrose Personal Research Fund, Sydney, Australia. The author has received consultancy fees from Arthropharm(Fidia) Pharmaceutical Company Ltd. who had no input into the writing of this review or the reason to publish.

## Conflicts of Interest

The author declares no conflicts of interest.

## Data Availability

The data that support the findings of this study are available on request from the corresponding author. The data are not publicly available due to privacy or ethical restrictions.
